# Trends of inequalities in childhood immunization coverage among children aged 12-23 months in Kenya, Ghana, and Côte d’Ivoire

**DOI:** 10.1186/s12889-019-7309-9

**Published:** 2019-07-23

**Authors:** Hermann Pythagore Pierre Donfouet, Gaye Agesa, Martin Kavao Mutua

**Affiliations:** 1 African Population and Health Research Center, APHRC Campus, 2nd Floor, Manga Close, Off Kirawa Road, P.O. Box: 10787-00100, Nairobi, Kenya; 2 University of Rennes, CNRS, CREM-UMR 6211, F-35000 Rennes, France

**Keywords:** Childhood immunization coverage, Inequality, Tailoring immunization programmes, D63, I1, O12

## Abstract

**Background:**

Immunization is one of the most cost-effective health intervention to halt the spread of childhood diseases, and improve child health. Yet, there is a substantial disparity in childhood immunization coverage. The overall objective of the study is to investigate the trends of within-country inequalities in childhood immunization coverage among children aged 12–23 months in Kenya, Ghana, and Côte d’Ivoire. The three countries included in this study are countries that are on the verge of entering the accelerated phase of the Gavi, the Vaccine Alliance’s co-sharing of costs of vaccine and eventually assuming full costs of vaccines. Côte d’Ivoire is in the Gavi preparatory transition phase, entering the accelerated transition phase in 2020, with an expected transition to full self-financing in 2025. Ghana is expected to enter the accelerated transition phase in 2021 and to full self-financing in 2026 while Kenya will enter in 2022 and fully self-finance in 2027.

We examine the pattern of inequality in childhood immunization coverage over time through an equity lens by mainly exploring the direction of inequality in coverage.

**Methods:**

We use data from the Demographic Health Surveys and Multiple Indicator Cluster Surveys. The rate difference, rate ratio, and relative concentration index are used as measures of inequality.

**Results:**

Results of the study suggest that in most years inequality in immunization coverage in the three countries persist over time, and it favors the most-advantaged households. However, there is a sharp decrease pattern in inequalities in childhood immunization coverage in Ghana over time.

**Conclusion:**

Policymakers could be more strategic in addressing pro-rich inequality in immunization coverage by designing health interventions through an equity lens. Using inequality data and putting disadvantaged households at the center of health intervention designs could increase the efficiency of the primary health care system and reduce the incidence of mortality and morbidity as a result of vaccine-preventable disease.

**Electronic supplementary material:**

The online version of this article (10.1186/s12889-019-7309-9) contains supplementary material, which is available to authorized users.

## Background

Over the past three decades, vaccines have been useful in reducing childhood mortality [1–4]. Each year immunization averts 2.5 million deaths from vaccine-preventable diseases (VPDs), in children younger than five years [5]. Given these benefits of vaccines, the Expanded Program on Immunization (EPI), which is committed to universal access to vaccines as a way to reduce incidence of childhood disease, has been implemented in all sub-Sahara African (SSA) countries. Since its introduction in 1978, this program has contributed to a reduction of diseases such as measles, polio and seen an increase in the coverage of childhood vaccines [6]. However, children from low socio-economic backgrounds or whose mothers have low level of education are missing these vital vaccines [7–12].

Immunization coverage is an important indicator in access and use of routine vaccinations. Although coverage has improved in most SSA countries, the within-country inequalities still exist. Inequality refers to the observed differences in coverage between different population subgroups. Measuring and monitoring these inequalities could be useful in designing health interventions that put the most-disadvantaged subgroups at the forefront. Furthermore, Gavi, the Vaccine Alliance has identified the need to monitor inequalities and this forms a core part of its policy [13, 14].

To motivate our empirical analysis, the conceptual framework of social determinants of health [15] is used to explain why inequality in immunization coverage could favor the most-advantaged subgroups. According to the theory of social determinants of health, factors such as social-cultural conditions and environmental context may influence individuals’ life and consequently, their actions towards disease prevention. Examples of social determinants include income, education which could explain these inequalities. Hence, wealthy and more educated mothers could be more knowledgeable about the benefits of vaccines and more inclined to have their children immunized. Individuals’ social position could, therefore, be the main driver of inequality [5].

To our knowledge, there are limited studies which investigate the trends of within-country inequalities in immunization coverage for children aged 12–23 months in Kenya, Ghana, and Côte d’Ivoire over time. The three countries included in this study are countries which are in the pipeline of exiting from Gavi’s financial support in 2027 (Kenya), 2026 (Ghana), and 2025 (Côte d’Ivoire). These countries are also part of the Immunization Advocacy Initiative (IAI) project. The IAI project is led by the African Population and Health Research Center and it aims at building the capacity of civil society organizations (CSOs) in countries which will soon transit from the Gavi support. Armed with technical skills and evidence on inequality in immunization coverage, these CSOs could be an effective lever of influence on government decisions to increase domestic financing for immunization and reduce equity in coverage of routine immunization.

This study uses data from cross-sectional Demographic Health Surveys (DHS) for each country between the years 1993 (or 1994) and the latest survey to examine the pattern of inequality in immunization coverage through an equity lens by mainly exploring the direction of inequality in immunization coverage over time. Knowledge of the variation of inequality may inform whether child health interventions implemented in different countries are associated with reduced inequality in immunization coverage. Furthermore, the analysis could help policymakers to develop immunization interventions through an equity lens. Another important contribution of the paper is the uncovering of the large variation in inequalities across countries. The fluctuations of inequalities across countries could provide important information for comparison. This could also shed more light on why some immunization interventions are more or less successful in curbing inequalities in different contexts.

In the next section, we describe the data source and measure of inequality in immunization coverage. In section 3, results are presented whereas in section 4, the findings are discussed with some policy recommendations. Section 5 concludes.

## Methods

### Data source

Data used in this study were obtained from the DHS covering the 1993–2014 period (Kenya and Ghana), DHS and Multiple Indicator Cluster Surveys (MICS) covering the 1994–2011 period (Côte d’Ivoire) and analyzed using the Health Equity Assessment Toolkit (HEAT). The periods differ by country due to data availability.

The HEAT [16] is a software developed by the World Health Organization (WHO) used to monitor health inequalities on 30 reproductive, maternal, newborn and child health indicators, disaggregated by five dimensions of inequality (economic status, education, place of residence, subnational region and child’s sex). It covers most of the low-and-middle-income countries. The DHS and MICS are uploaded in the HEAT. The DHS are nationally representative household survey conducted in low-and-middle-income countries for the purpose of monitoring and evaluating population, health and nutrition programs. It is a face-to-face survey on women aged 15–49 administered by highly trained enumerators. In DHS, specific questions were asked to women about children’s health. Questions related to immunization coverage are of particular interest. The enumerators either record the dates of different vaccines from the child health book/vaccination card or ask questions about whether or not the child has ever had some vaccines. We focus on routine vaccines among children aged 12–23 months. The DHS are implemented by ICF International and funded by the United States Agency for International Development. The MICS are household surveys that collect information on children under the age of five and women aged 15–49 in several countries. MICS are comparable to DHS and contain similar questions related to immunization coverage. MICS are managed by the United Nations Children’s Fund.

In the study, we use four immunization coverage indicators: Bacille Calmette-Guérin (BCG) coverage, diphtheria, tetanus toxoid and pertussis (DTP) coverage, measles coverage, poliomyelitis coverage, and full immunization coverage. BCG coverage is defined as the percentage of children aged 12–23 months who have received one dose of BCG vaccine given at birth, in a given year. DTP coverage is the percentage of children who have received three doses of the combined DTP vaccine given at age six, ten, and fourteen weeks respectively, in a given year. Measles coverage is defined as the percentage of children aged 12–23 months who have received at least one dose of measles-containing vaccine given in some countries at the age of nine months, in a given year. Poliomyelitis coverage is the percentage of children aged 12–23 months who have received three doses of polio vaccine given at age six, ten, and fourteen weeks respectively, in a given year. Full immunization coverage is the percentage of children aged 12–23 months who have received one dose of BCG vaccine, three doses of the polio vaccine, three doses of the combined DTP vaccine, and one dose of measles vaccine. Table 1 presents the definition of the indicators of immunization coverage.

**Table 1:** Immunization coverage indicators among children aged 12–23 months

Variables	Definitions
BCG coverage (bcgv)	The percentage of children who have received one dose of Bacille Calmette-Guérin (BCG) vaccine in a given year. The numerator is the number of children aged 12–23 months receiving one dose of BCG vaccine while the denominator is the total number of children aged 12–23 months surveyed.
DTP3 coverage (dptv)	The percentage of children who have received three doses of the combined diphtheria, tetanus toxoid, and pertussis (DTP3) vaccine in a given year. The numerator is the number of children aged 12–23 months receiving three doses of DTP3 vaccine while the denominator is total number of children aged 12–23 months surveyed.
Full immunization coverage (fullv)	The percentage of children who have received one dose of Bacille Calmette-Guérin (BCG) vaccine, three doses of polio vaccine, three doses of the combined diphtheria, tetanus toxoid and pertussis (DTP3) vaccine, and one dose of measles vaccine. The numerator is the number of children aged 12–23 months receiving one dose of BCG vaccine, three doses of polio vaccine, three doses of DTP3 vaccine, and one dose of measles vaccine while the denominator is the total number of children aged 12–23 months surveyed.
Measles coverage (mslv)	The percentage of children who have received at least one dose of measles-containing vaccine in a given year. The numerator is the number of children aged 12–23 months receiving at least one dose of measles-containing vaccine while the denominator is total number of children aged 12–23 months surveyed.
Polio coverage (poliov)	The percentage of children who have received three doses of polio vaccine in a given year. The numerator is the number of children aged 12–23 months receiving three doses of polio vaccine while the denominator is the total number of children aged 12–23 months surveyed.

Notes: Data sources are from DHS, MICS and analyzed using the HEAT software

### Inequality measures of ordered dimensions

Different statistics are used to measure inequality depending on whether the inequality dimensions are ordered or non-ordered [17]. Ordered dimensions such as economic status and education have an inherent ordering of subgroups, implying that households in the poorest quintile (with less education) have *less* of something compared to those with more wealth (more education). Non-ordered dimensions, by contrast, have subgroups that have no intrinsic ordering of subgroups such as gender, place of residence (urban vs. rural), subnational region. In this study, inequalities measures of ordered dimensions are used. The economic status of households was determined using a wealth index, which captures the households’ ownership of assets and access to some services. For each country selected, the wealth index was constructed using principal component analysis and households are classified into quintiles, ranging from the poorest quintile to the richest quintile. Additionally, we use mother’s education as another dimension of inequality. Education was an ordinal variable taking three categories: no education, primary education level, and secondary or higher.

In assessing the inequality in immunization coverage indicators, three inequality measures are used: rate difference (absolute inequality), rate ratio (relative inequality), and the relative concentration index. These measures are commonly used in the literature. The rate difference and rate ratio are simple measures of inequality which do not account for the population share and only looks at the extreme categories. Simply stated, the rate difference is the difference of immunization outcomes between the most-advantaged (richest quintile, secondary school or higher) and most-disadvantaged subgroups (poorest quintile, no education), whereas the rate ratio is the immunization outcomes in the most-advantaged (richest quintile, secondary school or higher) divided by the immunization outcomes in the most-disadvantaged subgroups (poorest quintile, no education). A positive value of the rate difference indicates that immunization coverage tends to favor the most-advantaged households. Similarly, a rate ratio greater than one means that immunization coverage tends to favor the most-advantaged households. The relative concentration index is a complex and sophisticated measure of inequality [18]. It accounts for the population share in different subgroups and is defined as twice the area between the line of equality and the concentration curve. It provides information on the extent to which immunization coverage is concentrated among the disadvantaged or the advantaged households. In the HEAT, the relative concentration index is bounded between − 100 and + 100 since it is multiplied by 100. Positive values indicate a concentration of the immunization coverage among the advantaged, while negative values indicate a concentration of the immunization coverage among the disadvantaged. We explore the significance of the relative concentration index using a *t-test* at the 5% level of significance. We also report the 95% confidence intervals in each figure for every country. The confidence intervals are estimated via bootstrap methods. The survey sampling design was taken into account for estimating all inequality measures.

Because our data cover several time points, we examine the pattern of inequality in immunization coverage over time through an equity lens by mainly exploring the variation of inequality in immunization coverage over time. Table 2 summarizes these inequality measures.

**Table 2:** Inequality measures of ordered dimensions

Inequality dimensions	Inequality measures	Definitions	Formula
Economic status Education	Rate difference (d)	Difference of immunization outcomes between the most-advantaged and most-disadvantaged subgroups.	*d* = *Y*_*max*_ − *Y*_*min*_
	Rate ratio (r)	Immunization outcomes in the most-advantaged divided by the immunization outcomes in the most-disadvantaged subgroups.	$r=\frac{Y_{max}}{Y_{min}}$
	Relative concentration index (rci)	It is twice the area between the line of equality and the concentration curve.	$rci=\left(\frac{\sum_j{P}_j\left(2{X}_j-1\right){Y}_j}{\mu}\right)\ast 100$

Notes: *Y*_*max*_, *Y*_*min*_ are the immunization outcomes of the most-advantaged subgroup, most-disadvantaged subgroup, respectively. *X*_*j*_, *P*_*j*_, *Y*_*j*_ are the relative rank, population share, and immunization outcomes of subgroup *j*, respectively. *μ* is the national average

## Results

### Main results

In Additional file 1: Table S1 we provide the coverage of childhood immunization over time for Kenya, Ghana, and Côte d’Ivoire. With regard to full immunization coverage, results suggest that over time, the three countries have not achieved the *Global Vaccine Action Plan* (GVAP) target goals of at least 90% national coverage. By focusing on each routine vaccine, the GVAP coverage target was not achieved by Côte d’Ivoire irrespective of the time period. For Kenya and Ghana, with regard to the DPT vaccine, the GVAP target goals of at least 90% national coverage were almost achieved only in 2014. However, these findings mask inequality in childhood immunization coverage over time.

#### Analysis of inequality in immunization coverage in Kenya

With regard to Kenya (Table 3), overall results suggest that immunization coverage favors the advantaged households over time irrespective of all inequality measures used except for the relative concentration index of polio in the year 2008 (− 0.04). The inequality in immunization coverage is persistent over time. A *t-test* on the coefficients of the relative concentration index of all immunization coverage indicators suggests that for the year 1993, all relative concentration indexes are positive and statistically different from zero. The same finding is valid for the year 1998 except for polio immunization. For the year 2003, the coefficients of the relative concentration index of all immunization coverage indicators are positive and statistically different from zero. In the same vein, for the year 2008, the coefficients of the relative concentration index of all immunization coverage indicators are positive and statistically different from zero except for BCG immunization, full immunization, and polio immunization. Results also suggest that for the year 2014, the coefficients of the relative concentration index of all immunization coverage indicators are positive and statistically different from zero except for polio immunization.

**Table 3:** Inequality in childhood immunization coverage using economic status as inequality dimension, Kenya

Economic status		Years									
		1993		1998		2003		2008		2014	
Indicators	Statistics	Value	SE	Value	SE	Value	SE	Value	SE	Value	SE
bcgv	d	5.77	2.29	5.57	1.96	26.20	3.98	3.74	2.47	5.60	1.39
	r	1.06	0.03	1.06	0.02	1.37	0.08	1.04	0.03	1.06	0.02
	rci	1.34	0.44	1.35	0.40	6.04	0.93	0.59	0.47	1.20	0.26
dptv	d	17.57	3.33	16.44	4.84	15.81	5.49	11.94	4.70	9.19	2.34
	r	1.23	0.05	1.24	0.08	1.28	0.11	1.15	0.07	1.11	0.03
	rci	3.96	0.74	4.26	1.12	5.55	1.44	2.58	1.00	2.14	0.47
fullv	d	21.59	4.26	11.82	6.12	20.37	5.41	7.98	5.68	11.16	3.98
	r	1.33	0.08	1.24	0.14	1.53	0.18	1.13	0.10	1.18	0.07
	rci	5.53	1.02	4.87	1.81	8.24	2.02	2.25	1.56	3.44	0.94
mslv	d	20.13	4.29	24.43	4.49	33.11	4.63	18.32	4.35	16.70	2.11
	r	1.29	0.07	1.38	0.09	1.60	0.12	1.24	0.07	1.22	0.03
	rci	4.23	0.93	5.70	1.06	9.13	1.34	4.46	0.99	4.07	0.44
poliov	d	17.84	3.32	7.93	5.20	11.21	5.44	1.85	5.35	1.51	3.61
	r	1.23	0.05	1.12	0.08	1.20	0.11	1.03	0.07	1.02	0.05
	rci	4.38	0.75	2.44	1.26	3.89	1.50	−0.04	1.31	0.43	0.76

Notes: bcgv, dptv, fullv, mslv, poliov are BCG immunization coverage, DTP3 immunization coverage, full immunization coverage, measles coverage and polio coverage among children aged 12–23 months, respectively. d, r and rci are rate difference, rate ratio and relative concentration index, respectively. SE is the standard errors. Economic status is used as the dimension of inequality. Economic status is determined using the wealth index. The wealth index is constructed on household assets and access to basic services using the principal component analysis. The wealth index is divided into quintiles. Data sources are from DHS, and analyzed using the HEAT software

**Fig. 1:**
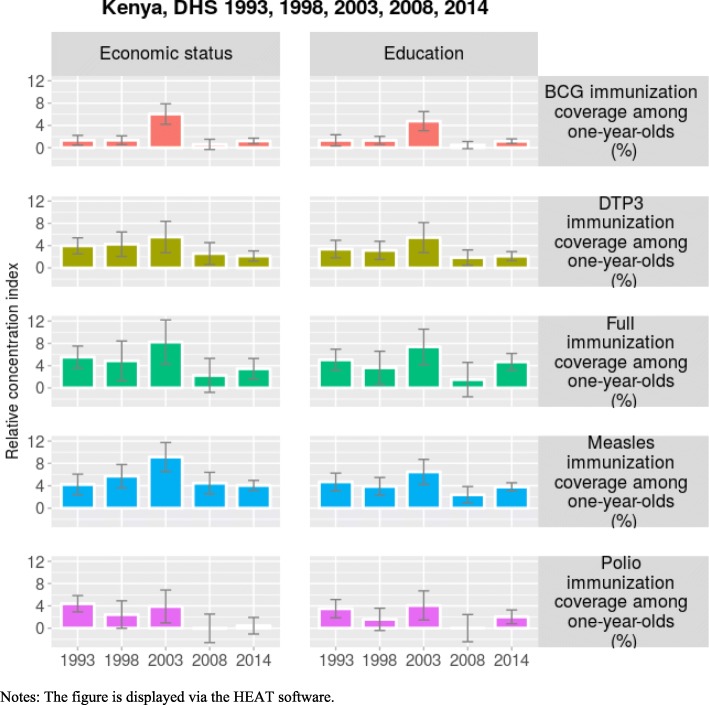
Trends in inequality in childhood immunization coverage, Kenya

In Fig. 1, over time inequality in immunization coverage in Kenya follows an inverted U-shape curve except for polio immunization. The turning point occurs in 2003. This result suggests an improvement of the equity of immunization coverage, indicating that with time the Kenyan government has been aware of inequality in immunization coverage and endeavored to reduce it. The 95% confidence intervals displayed in Fig. 1 corroborates the findings drawn on the significance of the coefficients of the relative concentration index when a *t-test* is used.

#### Analysis of inequality in immunization coverage in Ghana

**Fig. 2:**
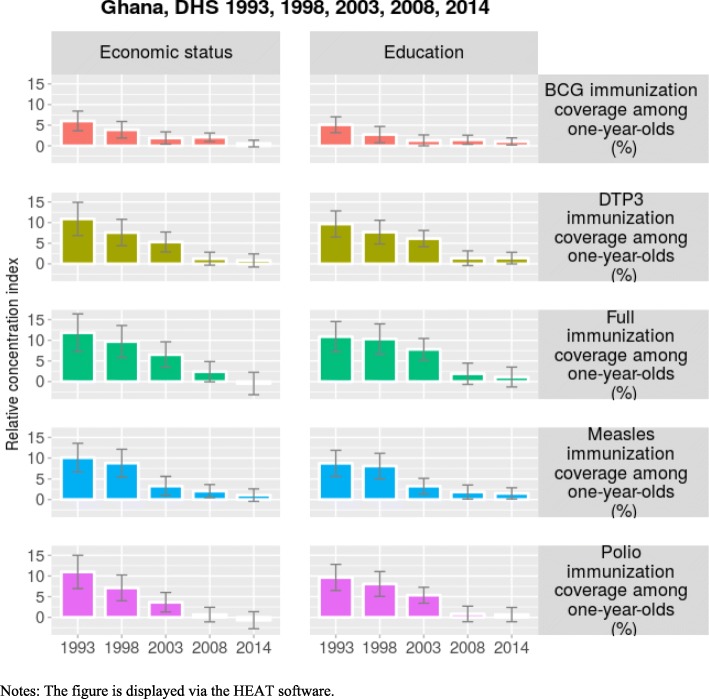
Trends in inequality in childhood in childhood immunization coverage, Ghana

Table 4 shows that in most cases, inequality in immunization coverage in Ghana favors the advantaged households. A *t-test* on the coefficients of the relative concentration index of all immunization coverage coefficients indicates that inequality in immunization coverage is positive and statistically different from zero in most years. Figure 2 indicates a decreasing pattern of inequality in immunization coverage over time. It seems over time, the Ghanaian government has consistently shrunk inequality in immunization coverage.

**Table 4:** Inequality in childhood immunization coverage using economic status as inequality dimension, Ghana

Economic status		Years									
		1993		1998		2003		2008		2014	
Indicators	Statistics	Value	SE	Value	SE	Value	SE	Value	SE	Value	SE
bcgv	d	28.09	5.42	17.74	4.44	7.64	3.84	9.29	2.84	3.16	2.84
	r	1.42	0.11	1.23	0.07	1.09	0.05	1.10	0.03	1.03	0.03
	rci	6.04	1.22	3.91	1.02	1.91	0.76	2.07	0.54	0.57	0.54
dptv	d	39.95	6.80	31.54	5.77	21.69	5.27	4.14	4.17	3.83	4.17
	r	1.92	0.26	1.52	0.13	1.33	0.09	1.05	0.05	1.04	0.05
	rci	10.88	2.06	7.59	1.63	5.29	1.23	1.25	0.80	0.82	0.80
fullv	d	41.67	6.73	32.26	6.35	24.70	5.86	8.09	5.76	−2.57	5.76
	r	2.11	0.32	1.66	0.18	1.45	0.13	1.11	0.08	0.97	0.08
	rci	11.83	2.33	9.70	1.98	6.54	1.57	2.40	1.25	−0.47	1.25
mslv	d	41.37	5.69	29.57	6.10	13.83	5.16	7.23	4.03	6.41	4.03
	r	1.87	0.20	1.50	0.14	1.18	0.08	1.08	0.05	1.07	0.05
	rci	10.09	1.77	8.76	1.72	3.27	1.17	2.06	0.81	1.08	0.81
poliov	d	39.95	6.80	26.81	5.94	15.47	5.20	2.56	4.55	−4.30	4.55
	r	1.92	0.26	1.44	0.12	1.23	0.09	1.03	0.05	0.95	0.05
	rci	10.98	2.06	7.12	1.59	3.65	1.21	0.66	0.90	−0.71	0.90

Notes: bcgv, dptv, fullv, mslv, poliov are BCG immunization coverage, DTP3 immunization coverage, full immunization coverage, measles coverage and polio coverage among children aged 12–23 months, respectively. d, r and rci are rate difference, rate ratio and relative concentration index, respectively. SE is the standard errors. Economic status is determined using the wealth index. The wealth index is constructed on household assets and access to basic services using the principal component analysis. The wealth index is divided into quintiles. Data sources are from DHS, and analyzed using the HEAT software

#### Analysis of inequality in immunization coverage in Côte d’Ivoire

**Fig. 3:**
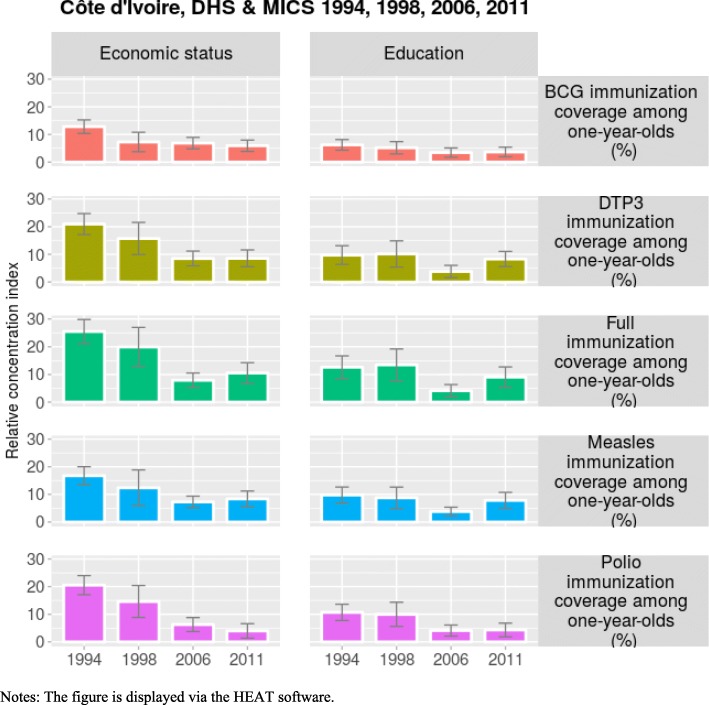
Trends in inequality in childhood in childhood immunization coverage, Côte d’Ivoire

In most years there is a pro-rich inequality in immunization coverage in Côte d’Ivoire (Table 5). Because the 95% confidence intervals do not contain zero, it is relevant to ascertain that the coefficients of the relative concentration index in immunization coverage is positive and statistically different from zero in all years (Fig. 3). Furthermore, in most years there is a decreasing pattern of inequality in immunization coverage over time. Hence, though there is still a pro-rich inequality in immunization coverage in Côte d’Ivoire, this inequality is reduced over time.

**Table 5:** Inequality in childhood immunization coverage using economic status as inequality dimension, Côte d’Ivoire

Economic status		Years							
		1994		1998		2006		2011	
Indicators	Statistics	Value	SE	Value	SE	Value	SE	Value	SE
bcgv	d	46.74	4.26	31.85	6.71	27.80	4.36	23.85	4.28
	r	1.96	0.16	1.47	0.15	1.39	0.08	1.33	0.08
	rci	12.83	1.24	7.27	1.80	6.88	1.06	5.90	1.05
dptv	d	47.96	5.02	51.45	7.63	34.13	5.20	28.56	5.44
	r	2.85	0.40	2.33	0.43	1.55	0.13	1.55	0.14
	rci	20.95	1.94	15.72	2.97	8.49	1.37	8.55	1.56
fullv	d	48.44	4.55	55.11	7.51	31.20	5.19	28.82	5.45
	r	4.08	0.74	2.85	0.64	1.50	0.12	1.73	0.18
	rci	25.48	2.21	19.92	3.58	7.91	1.33	10.52	1.92
mslv	d	47.80	4.46	48.95	9.23	29.13	4.34	29.19	4.82
	r	2.55	0.28	2.06	0.40	1.42	0.09	1.54	0.12
	rci	16.75	1.67	12.37	3.31	7.26	1.06	8.36	1.45
poliov	d	51.68	4.77	47.95	7.84	26.29	5.38	15.65	5.15
	r	2.84	0.38	2.19	0.39	1.39	0.11	1.24	0.09
	rci	20.53	1.76	14.62	2.95	6.26	1.30	3.97	1.34

Notes: bcgv, dptv, fullv, mslv, poliov are BCG immunization coverage, DTP3 immunization coverage, full immunization coverage, measles coverage and polio coverage among children aged 12–23 months, respectively. d, r and rci are rate difference, rate ratio and relative concentration index, respectively. SE is the standard errors. Economic status is determined using the wealth index. The wealth index is constructed on household assets and access to basic services using the principal component analysis. The wealth index is divided into quintiles. Data sources are from DHS and MICS, and analyzed using the HEAT software

### Sensitivity analysis

We explore the robustness of the findings using education as the dimension of inequality. In Kenya, results indicate that inequality in immunization coverage persists over time and favors the advantaged households (Additional file 1: Table S2). In most years, a *t-test* on the relative concentration index of all immunization coverage coefficients, suggests that inequality in immunization coverage is positive and statistically different from zero. Furthermore, Fig. 1 suggests an inverted U-shape curve except for polio immunization. The findings are similar to Ghana and Côte d’Ivoire except that inequality in immunization coverage decreases over time (Additional file 1: Table S3 and Table S4).

## Discussions

In most years, this study shows that inequality in immunization coverage in Kenya, Ghana, and Côte d’Ivoire is persistent over time, and it favors the most-advantaged households. This result could be explained by the fact that the most-advantaged households could be more informed about the benefits of immunization/vaccines which in turn increases their demand for immunization. They could also have more access to health facilities offering immunization services. In Kenya, over time we observe an inverted U-shape curve in inequality in immunization coverage except for polio immunization with the turning occurring in 2003. Results from Ghana suggest a decreasing pattern of inequality in immunization coverage over time. This result suggests that policymakers in these countries seem to have deployed some laudable efforts to reach the disadvantaged households, and thus reduce inequality though it still a concern. Our findings are similar to Restrepo-Méndez et al. [7]‘s study who found a pro-rich inequality in full immunization coverage in some low-and-middle-income countries though inequality in immunization coverage over time and its pattern in Kenya, Ghana, and Côte d’Ivoire were not explored.

Furthermore, the different shapes of inequality in immunization coverage over time and space raise some questions: what explains the differences over space and time? All three countries benefited from Gavi financial support. Why were these interventions more or less successful in curbing inequalities in different contexts? For instance, using the most recent estimates on full immunization for year 2014, Kenya and Ghana have no major difference in full immunization coverage (the median value is 73.2, 78.6%, for Kenya and Ghana, respectively) but very different shapes of inequality over time: monotonic decrease in Ghana, and inverted U-shape in Kenya. We argue that several factors could explain this finding. First, the role and actions of CSOs in these countries could explain this variation since they are considered by Gavi as key actors in delivering health services and immunization to the remote areas and disadvantaged households. It is well established in the literature that CSOs can be used as a tool to address inequality [19, 20]. Hence, in countries with weak CSOs, public health spending meant for immunization could be more profitable to the most-advantaged households. An inspection of the Civil Society Organization Sustainability Index (CSOSI) published by the USAID for Kenya and Ghana for the most recent years suggest that the two countries are within the sustainability evolving category. But, the CSOSI do not shed more lights on the classification of CSOs in each country with regard to their advocacy capacity to immunization. This indicates that more research is needed to explore the link between the CSOs immunization advocacy and inequality in immunization. Second, shifting responsibilities in health from higher to lower levels could also explain this variation. In Kenya and Ghana, from our interaction with CSOs working in these countries, the community health volunteers (CHVs) seem to be more functional than in Côte d’Ivoire in terms of linking communities to health facilities and regulation. These CHVs provide vital health services to their communities, enhance immunization coverage, and in some Kenyan counties, they are currently being considered as part of the formal healthcare system. It is not therefore surprising that inequality in immunization in Côte d’Ivoire is much higher than in Kenya and Ghana. For instance, the CHVs have been major players in the delivery of essential health service since the 1980s in Kenya and the country adopted the community health strategy in 2007. Concerning Ghana, it is difficult to trace when the CHVs emerged. But, in the literature [21, 22] CHVs were spearheaded by faith-based organizations namely in 1999 where a community-based project was established to provide essential health services. Given the success of this project, in 2005 the Ghanaian government adopted and named it as the community-based health planning and services (CHPS) programme as a national health policy [23]. In Côte d’Ivoire, CHVs still provide health promotion services but they are unregulated because there are no clear national guidelines governing their roles [24]. Third, total expenditures on routine immunization from all sources per capita ($US) could also explain this variation. As displayed in Additional file 1: Table S5, using more recent estimates on total expenditures on routine immunization from all sources per capita, in most years Ghana has been spending more on routine immunization per capita than Kenya and Côte d’Ivoire. Fourth, the sharp decrease of inequalities in immunization coverage in Ghana could be explained by the adoption a more ambitious health reform such as the National Health Insurance Scheme (NHIS) aimed at providing equitable access and financial protection for healthcare services to Ghanaian citizens. As outlined by Grépin and Dionne [25], the adoption of NHIS in Ghana was more comprehensive and inclusive since it called for universal coverage and a health package that covered nearly the entire disease burden present in the country. In Ghana, the share of immunization delivery costs is implicitly covered by NHIS payments to healthcare providers. In the same vein, the Ghanaian government has been a strong voice and advocate of child health and strong political will to Gavi’s mission. For instance, John Mahama (president from 2012 to 2017) contributed to the co-hosting of Gavi’s mid-term review in 2013.

The study has several policy implications. First, in Kenya, Ghana and Côte d’Ivoire, policymakers could be more strategic in addressing pro-rich inequality in immunization coverage by designing health interventions through an equity lens. Though reaching every district is already implemented, the Tailoring Immunization Programmes (TIP) to reach the disadvantaged households could be an effective strategy. The TIP [26, 27] aims at: (i) identifying households susceptible to vaccine-preventable diseases; (ii) diagnosing supply-and demand-side barriers and motivators to vaccination; and (iii) recommending evidence-informed responses to sustain vaccination. Therefore, the TIP could help policymakers in those countries to involve all stakeholders, analyze the barriers to vaccination, research and design customized solutions/strategies to increase immunization coverage of disadvantaged households, implement and monitor these customized solutions/strategies. Another prominent strategy is the adoption of a health system reform that is more inclusive and investing more in immunization services with the aim of reaching more the underserved population. It seems this has been the case for Ghana and it is not surprising that there is a sharp decrease pattern in inequalities in childhood immunization coverage. Second, results suggest that for improvement in coverage and equitable access to immunization, measuring and monitoring of immunization coverage should be integrated into the country health strategic plan. This could ensure that in terms of immunization coverage no child is left behind. The major limitation of the paper is that the concentration index is not decomposed to identify factors that could explain the concentration of immunization coverage among the most-advantaged households over time.

## Conclusions

The main results of the study suggest that in most years inequality in immunization coverage in the three countries persist over time, and it favors the most-advantaged households. Policymakers could be more strategic in addressing pro-rich inequality in immunization coverage by designing health interventions through an equity lens. Tailoring immunization programmes to reach the disadvantaged households could be an effective strategy. Our findings suggest that investing more in immunization services with the aim of reaching more the underserved population combined with a strong political could be a promising strategy.

However, the current study has some limitations. First, the study does not fully explain the childhood immunization coverage over time. We could have decomposed the estimated concentration index to identify factors that explain the concentration of childhood immunization coverage among the rich over time. Second, the effect of migration policies in each country is not explored.

## Additional file

Additional file 1: **Table S1.** Coverage of childhood immunization over time. **Table S2.** Inequality in childhood immunization coverage using education as inequality dimension, Kenya. **Table S3.** Inequality in childhood immunization coverage using education as inequality dimension, Ghana. **Table S4.** Inequality in childhood immunization coverage using education as inequality dimension, Côte d’Ivoire. **Table S5.** Comparison of the total expenditures on routine immunization from all sources (US$ per capita) over time. (DOCX 45 kb)

## Data Availability

All data used and analyzed during the current study are publicly available from the Demographic Health Survey. The data sets are found/uploaded in the Health Equity Assessment Toolkit (HEAT). The findings can be replicated using the HEAT available at this link: https://whoequity.shinyapps.io/HEAT/

## Abbreviations

BCG: Bacille Calmette-Guérin
CSOs: Civil society organizations
DHS: Demographic Health Surveys
DTP: Diphtheria, tetanus toxoid and pertussis
EPI: Expanded Program on Immunization
*GVAP*: The *Global Vaccine Action Plan*
HEAT: Health Equity Assessment Toolkit
IAI: Immunization Advocacy Initiative
MICS: Multiple Indicator Cluster Surveys
VPDs: Vaccine-preventable diseases

## References

[1] McGovern ME, Canning D (2015). Vaccination and all-cause child mortality from 1985 to 2011: global evidence from the demographic and health surveys. Am J Epidemiol.

[2] Aaby P, Bukh J, Lisse IM, Smits AJ (1984). Measles vaccination and reduction in child mortality: a community study from Guinea-Bissau. J Infect.

[3] Koenig MA, Khan MA, Wojtyniak B, Clemens JD, Chakraborty J, Fauveau V (1990). Impact of measles vaccination on childhood mortality in rural Bangladesh. Bull World Health Organ.

[4] Higgins JP, Soares-Weiser K, López-López JA, Kakourou A, Chaplin K, Christensen H (2016). Association of BCG, DTP, and measles containing vaccines with childhood mortality: systematic review. BMJ..

[5] Bryce J, Black RE, Walker N, Bhutta ZA, Lawn JE, Steketee RW (2005). Can the world afford to save the lives of 6 million children each year?. Lancet.

[6] Schlumberger M, Bamoko A, Yameogo T, Rouvet F, Ouedraogo R, Traore B (2015). Positive impact on the expanded program on immunization when sending call-back SMS through a computerized immunization register, Bobo Dioulasso (Burkina Faso). Bulletin de la Société de Pathologie Exotique (1990).

[7] Restrepo-Méndez MC, Barros AJ, Wong KL, Johnson HL, Pariyo G, França GV (2016). Inequalities in full immunization coverage: trends in low-and middle-income countries. Bull World Health Organ.

[8] Gram L, Soremekun S, ten Asbroek A, Manu A, O'leary M, Hill Z (2014). Socio-economic determinants and inequities in coverage and timeliness of early childhood immunisation in rural Ghana. Tropical Med Int Health.

[9] Schoeps A, Ouedraogo N, Kagone M, Sie A, Müller O, Becher H (2013). Socio-demographic determinants of timely adherence to BCG, Penta3, measles, and complete vaccination schedule in Burkina Faso. Vaccine..

[10] Özer M, Fidrmuc J, Eryurt MA (2018). Maternal education and childhood immunization in Turkey. Health Econ.

[11] Vikram K, Vanneman R, Desai S (2012). Linkages between maternal education and childhood immunization in India. Soc Sci Med.

[12] Abuya B, Onsomu E, Kimani J, Moore D (2011). Influence of maternal education on child immunization and stunting in Kenya. Matern Child Health J.

[13] Gandhi G (2015). Charting the evolution of approaches employed by the global Alliance for vaccines and immunizations (GAVI) to address inequities in access to immunization: a systematic qualitative review of GAVI policies, strategies and resource allocation mechanisms through an equity lens (1999–2014). BMC Public Health.

[14] Arsenault C, Harper S, Nandi A, Rodríguez JMM, Hansen PM, Johri M (2017). An equity dashboard to monitor vaccination coverage. Bull World Health Organ.

[15] WHO (2008). Commission on social determinants of health: final report.

[16] Hosseinpoor AR, Nambiar D, Schlotheuber A, Reidpath D, Ross Z (2016). Health equity assessment toolkit (HEAT): software for exploring and comparing health inequalities in countries. BMC Med Res Methodol.

[17] HEAT (2018). Software for exploring and comparing health inequalities in countries. Built-in database edition.

[18] WHO (2015). State of inequality: reproductive maternal newborn and child health: interactive visualization of health data.

[19] Bernhard M, Jung D-J (2017). Civil society and income inequality in post-communist Eurasia. Comparative Politics.

[20] Ekiert G, Kubik J, Wenzel M (2017). Civil society and three dimensions of inequality in post-1989 Poland. Comparative Politics..

[21] Nyonator FK, Awoonor-Williams JK, Phillips JF, Jones TC, Miller RA (2005). The Ghana community-based health planning and services initiative for scaling up service delivery innovation. Health Policy Plan.

[22] Aikins A-G, Koram K, Aryeetey E, Kanbur R (2017). Health and healthcare in Ghana. The economy of Ghana sixty years after independence.

[23] Adongo PB, Tapsoba P, Phillips JF, Tabong PT-N, Stone A, Kuffour E (2013). The role of community-based health planning and services strategy in involving males in the provision of family planning services: a qualitative study in southern Ghana. Reprod Health.

[24] Muriuki AM, Moss T (2016). The impact of Para-professional social workers and community health care workers in Côte d'Ivoire: contributions to the protection and social support of vulnerable children in a resource poor country. Child Youth Serv Rev.

[25] Grépin KA, Dionne KY (2013). Democratization and universal health coverage: a case comparison of Ghana, Kenya, and Senegal. Global Health Governance.

[26] Butler R, MacDonald NE (2015). Diagnosing the determinants of vaccine hesitancy in specific subgroups: the guide to tailoring immunization Programmes (TIP). Vaccine..

[27] WHO. The guide to tailoring immunization programmes (TIP). Copenhagen, Copenhagen, Denmark: World Health Organization; 2013.

