# Supplementary material for: Trends of inequalities in childhood immunization coverage among children aged 12-23 months in Kenya, Ghana, and Côte d’Ivoire
Source: BMC Public Health. 2019 Jul 23;19:988. doi: 10.1186/s12889-019-7309-9 (PMC6651994; doi:10.1186/s12889-019-7309-9)
Supplement: Supplementary file 1 — Table S1. Coverage of childhood immunization over time. Table S2. Inequality in childhood immunization coverage using education as inequality dimension, Kenya. Table S3. Inequality in childhood immunization coverage using education as inequality dimension, Ghana. Table S4. Inequality in childhood immunization coverage using education as inequality dimension, Côte d’Ivoire. Table S5. Comparison of the total expenditures on routine immunization from all sources (US$ per capita) over time. (DOCX 45 kb) [file 12889_2019_7309_MOESM1_ESM.docx]

**Table S1: Coverage of childhood immunization over time**

|  | Panel A: Coverage for Kenya and Ghana | | | | | |
| --- | --- | --- | --- | --- | --- | --- |
|  | **Years** | **1993** | **1998** | **2003** | **2008** | **2014** |
| bcgv | Kenya | 96.2 | 95.9 | 87.3 | 95.6 | 96.7 |
|  | Ghana | 83.1 | 87.8 | 91.1 | 95.8 | 96.8 |
| dptv | Kenya | 87 | 79.4 | 72.7 | 86.6 | 90.1 |
|  | Ghana | 62.4 | 72.2 | 79.8 | 89.3 | 89 |
| fullv | Kenya | 78.3 | 59.7 | 52 | 68.6 | 71.3 |
|  | Ghana | 54.8 | 62 | 69.6 | 79.4 | 78.2 |
| mslv | Kenya | 83.8 | 79.2 | 72.5 | 85 | 87.1 |
|  | Ghana | 64.4 | 72.6 | 83.2 | 90.2 | 89.3 |
| poliov | Kenya | 86 | 74.4 | 67.2 | 77.2 | 81.5 |
|  | Ghana | 62.4 | 71.6 | 79.6 | 86.9 | 85.4 |
|  | **Panel B: Coverage for Côte d’Ivoire** | | | | | |
|  | **Years** | **1994** | **1998** | **2006** | **2011** | |
| bcgv | Côte d’Ivoire | 73.8 | 83.7 | 85.9 | 83.4 | |
| dptv |  | 46.3 | 60.9 | 79.3 | 63.8 | |
| fullv |  | 37.4 | 50.7 | 77.5 | 50.5 | |
| mslv |  | 53.1 | 66.2 | 84.5 | 64.5 | |
| poliov |  | 51.4 | 60.6 | 81.6 | 69.2 | |

Notes: bcgv, dptv, fullv, mslv, poliov, are BCG immunization coverage, DTP3 immunization coverage, full immunization coverage, measles coverage and polio coverage among children aged 12-23 months, respectively. The periods differ by country due to data availability.

**Table S2: Inequality in childhood immunization coverage using education as inequality dimension, Kenya**

| Education | | Years | | | | | | | | | |
| --- | --- | --- | --- | --- | --- | --- | --- | --- | --- | --- | --- |
|  |  | **1993** | | **1998** | | **2003** | | **2008** | | **2014** | |
| Indicators | **Statistics** | **Value** | **SE** | **Value** | **SE** | **Value** | **SE** | **Value** | **SE** | **Value** | **SE** |
| bcgv | d | 8.96 | 3.36 | 10.61 | 3.27 | 32.60 | 5.73 | 3.18 | 2.43 | 8.84 | 1.46 |
|  | r | 1.10 | 0.04 | 1.12 | 0.04 | 1.53 | 0.14 | 1.03 | 0.03 | 1.10 | 0.02 |
|  | rci | 1.32 | 0.52 | 1.31 | 0.37 | 4.77 | 0.88 | 0.47 | 0.33 | 1.16 | 0.21 |
| dptv | d | 19.42 | 4.63 | 18.40 | 5.28 | 29.22 | 7.25 | 10.56 | 4.86 | 15.69 | 2.69 |
|  | r | 1.26 | 0.08 | 1.27 | 0.10 | 1.55 | 0.20 | 1.13 | 0.07 | 1.20 | 0.04 |
|  | rci | 3.37 | 0.81 | 3.15 | 0.83 | 5.44 | 1.37 | 1.86 | 0.70 | 2.12 | 0.42 |
| fullv | d | 25.13 | 4.84 | 15.14 | 7.10 | 27.96 | 5.81 | 5.91 | 7.87 | 25.88 | 3.45 |
|  | r | 1.40 | 0.10 | 1.30 | 0.17 | 1.83 | 0.28 | 1.09 | 0.12 | 1.50 | 0.09 |
|  | rci | 5.04 | 0.97 | 3.62 | 1.51 | 7.37 | 1.62 | 1.50 | 1.57 | 4.67 | 0.78 |
| mslv | d | 24.95 | 4.35 | 19.62 | 4.97 | 33.89 | 5.88 | 13.23 | 5.23 | 23.28 | 2.33 |
|  | r | 1.36 | 0.08 | 1.28 | 0.09 | 1.66 | 0.18 | 1.17 | 0.08 | 1.33 | 0.04 |
|  | rci | 4.68 | 0.80 | 3.89 | 0.80 | 6.48 | 1.14 | 2.40 | 0.75 | 3.79 | 0.37 |
| poliov | d | 20.08 | 4.74 | 10.47 | 5.90 | 21.21 | 6.49 | 0.10 | 6.51 | 14.84 | 3.28 |
|  | r | 1.28 | 0.08 | 1.16 | 0.10 | 1.42 | 0.17 | 1.00 | 0.08 | 1.21 | 0.05 |
|  | rci | 3.50 | 0.84 | 1.58 | 1.02 | 4.07 | 1.33 | 0.00 | 1.24 | 2.02 | 0.63 |

Notes: bcgv, dptv, fullv, mslv, poliov are BCG immunization coverage, DTP3 immunization coverage, full immunization coverage, measles coverage and polio coverage among children aged 12-23 months, respectively. d, r and rci are rate difference, rate ratio and relative concentration index, respectively. SE is the standard errors. Education is used as the dimension of inequality. **Education** refers to the highest level of schooling attained by the mothers/caregivers. It takes three categories: no education, primary school, secondary school or higher. Data sources are from DHS, and analyzed using the HEAT software.

**Table S3: Inequality in childhood immunization coverage using education as inequality dimension, Ghana**

| Education | | Years | | | | | | | | | |
| --- | --- | --- | --- | --- | --- | --- | --- | --- | --- | --- | --- |
|  | | **1993** | | **1998** | | **2003** | | **2008** | | **2014** | |
| Indicators | **Statistics** | **Value** | **SE** | **Value** | **SE** | **Value** | **SE** | **Value** | **SE** | **Value** | **SE** |
| bcgv | d | 26.56 | 2.97 | 10.15 | 3.66 | 4.92 | 2.60 | 6.73 | 2.43 | 5.18 | 2.07 |
|  | r | 1.36 | 0.06 | 1.12 | 0.05 | 1.06 | 0.03 | 1.07 | 0.03 | 1.06 | 0.02 |
|  | rci | 5.11 | 0.98 | 2.73 | 1.00 | 1.33 | 0.67 | 1.48 | 0.56 | 1.08 | 0.44 |
| dptv | d | 41.89 | 6.11 | 23.46 | 4.29 | 20.48 | 3.32 | 5.76 | 3.57 | 4.43 | 2.90 |
|  | r | 1.85 | 0.16 | 1.40 | 0.09 | 1.30 | 0.06 | 1.07 | 0.04 | 1.05 | 0.03 |
|  | rci | 9.65 | 1.61 | 7.68 | 1.46 | 6.12 | 1.01 | 1.37 | 0.90 | 1.40 | 0.72 |
| fullv | d | 44.48 | 6.70 | 27.00 | 4.68 | 22.82 | 3.91 | 7.12 | 4.58 | 3.38 | 4.30 |
|  | r | 2.05 | 0.21 | 1.57 | 0.14 | 1.39 | 0.08 | 1.10 | 0.07 | 1.04 | 0.06 |
|  | rci | 10.88 | 1.86 | 10.30 | 1.87 | 7.80 | 1.34 | 1.89 | 1.32 | 1.12 | 1.23 |
| mslv | d | 40.99 | 5.13 | 24.94 | 4.57 | 11.14 | 3.38 | 7.14 | 3.47 | 5.74 | 2.81 |
|  | r | 1.78 | 0.14 | 1.43 | 0.10 | 1.14 | 0.05 | 1.08 | 0.04 | 1.07 | 0.03 |
|  | rci | 8.72 | 1.60 | 8.07 | 1.56 | 3.25 | 0.96 | 1.83 | 0.87 | 1.49 | 0.69 |
| poliov | d | 41.89 | 6.11 | 24.51 | 4.43 | 17.74 | 3.27 | 3.54 | 3.67 | 1.11 | 3.33 |
|  | r | 1.85 | 0.16 | 1.43 | 0.10 | 1.25 | 0.05 | 1.04 | 0.04 | 1.01 | 0.04 |
|  | rci | 9.65 | 1.61 | 8.07 | 1.53 | 5.34 | 0.98 | 0.81 | 0.96 | 0.65 | 0.88 |

Notes: bcgv, dptv, fullv, mslv, poliov are BCG immunization coverage, DTP3 immunization coverage, full immunization coverage, measles coverage and polio coverage among children aged 12-23 months, respectively. d, r and rci are rate difference, rate ratio and relative concentration index, respectively. SE is the standard errors. Education is used as the dimension of inequality. **Education** refers to the highest level of schooling attained by the mothers/caregivers. It takes three categories: no education, primary school, secondary school or higher. Data sources are from DHS, and analyzed using the HEAT software [16].

**Table S4: Inequality in childhood immunization coverage using education as inequality dimension, Côte d’Ivoire**

| Education | | Years | | | | | | | |
| --- | --- | --- | --- | --- | --- | --- | --- | --- | --- |
|  |  | **1994** | | **1998** | | **2006** | | **2011** | |
| Indicators | **Statistics** | **Value** | **SE** | **Value** | **SE** | **Value** | **SE** | **Value** | **SE** |
| bcgv | d | 28.71 | 3.18 | 22.79 | 3.32 | 14.75 | 3.38 | 16.20 | 3.55 |
|  | r | 1.43 | 0.06 | 1.30 | 0.06 | 1.18 | 0.05 | 1.21 | 0.05 |
|  | rci | 6.23 | 0.98 | 5.17 | 1.13 | 3.44 | 0.86 | 3.67 | 0.88 |
| dptv | d | 33.00 | 4.75 | 44.11 | 4.87 | 14.73 | 4.39 | 28.69 | 4.63 |
|  | r | 1.81 | 0.14 | 1.85 | 0.15 | 1.20 | 0.06 | 1.51 | 0.10 |
|  | rci | 9.73 | 1.72 | 10.12 | 2.42 | 3.79 | 1.14 | 8.31 | 1.40 |
| fullv | d | 31.81 | 4.92 | 47.89 | 7.20 | 14.89 | 4.54 | 27.27 | 5.13 |
|  | r | 2.03 | 0.20 | 2.17 | 0.26 | 1.21 | 0.07 | 1.62 | 0.14 |
|  | rci | 12.63 | 2.10 | 13.47 | 2.93 | 4.11 | 1.14 | 9.03 | 1.89 |
| mslv | d | 34.64 | 4.36 | 36.87 | 6.51 | 17.72 | 2.93 | 29.97 | 4.41 |
|  | r | 1.76 | 0.12 | 1.64 | 0.14 | 1.22 | 0.04 | 1.53 | 0.10 |
|  | rci | 9.73 | 1.49 | 8.76 | 1.98 | 3.78 | 0.80 | 7.84 | 1.48 |
| poliov | d | 40.32 | 4.14 | 42.22 | 5.18 | 15.44 | 4.12 | 11.12 | 5.10 |
|  | r | 1.92 | 0.12 | 1.81 | 0.15 | 1.20 | 0.06 | 1.17 | 0.08 |
|  | rci | 10.69 | 1.51 | 9.98 | 2.23 | 4.11 | 1.02 | 4.31 | 1.26 |

Notes: bcgv, dptv, fullv, mslv, poliov are BCG immunization coverage, DTP3 immunization coverage, full immunization coverage, measles coverage and polio coverage among children aged 12-23 months, respectively. d, r and rci are rate difference, rate ratio and relative concentration index, respectively. SE is the standard errors. Education is used as the dimension of inequality. **Education** refers to the highest level of schooling attained by the mothers/caregivers. It takes three categories: no education, primary school, secondary school or higher. Data sources are from DHS and MICS, and analyzed using the HEAT software [16].

**Table S5: Comparison of the total expenditures on routine immunization from all sources (US$ per capita) over time**

| Variables | Years | 2010 | 2011 | 2012 | 2013 | 2014 | 2015 |
| --- | --- | --- | --- | --- | --- | --- | --- |
| Total expenditures on routine immunization from all sources (US$ per capita) | Ghana | 2.025 | 1.742 | 1.472 | 1.215 | 1.032 | 1.231 |
|  | Kenya | 0.182 | 0.0004 | 0.007 | 0.898 | 0.856 | 0.816 |
|  | Côte d’Ivoire | 0.515 | 0.515 | 0.538 | 0.504 | 1.281 | 0.976 |
| Difference in total expenditures on routine immunization from all sources (US$ per capita) between countries | Ghana vs. Kenya | 1.843 | 1.742 | 1.465 | 0.317 | 0.176 | 0.415 |
|  | Ghana vs. Côte d’Ivoire | 1.51 | 1.227 | 0.934 | 0.711 | -0.249 | 0.255 |
|  | Kenya vs. Côte d’Ivoire | -0.333 | -0.515 | -0.531 | 0.394 | -0.425 | -0.16 |

Sources: Total expenditures on routine immunization from all sources (US$ per capita) are computed by dividing the total expenditures on routine immunization from all sources by the total population. Data on total expenditures on routine immunization from all sources and total population are extracted from the WHO/UNICEF-JRF database. World Bank Development Indicator, respectively. Missing values from the WHO/UNICEF-JRF database were computed via linear interpolation.
